# Letter to the Editor: A reply – acknowledged reasonable limitations in a secondary analysis but key conclusions remain in ‘The neural basis of flashback formation: the impact of viewing trauma’

**DOI:** 10.1017/S0033291716000052

**Published:** 2016-03-11

**Authors:** I. A. Clark, C. E. MacKay, E. A. Holmes, C. Bourne

**Affiliations:** 1 Wellcome Trust Centre for Neuroimaging, Institute of Neurology, University College London, 12 Queen Square, London WC1N 3BG, UK; 2 University of Oxford Department of Psychiatry, Warneford Hospital, Oxford OX3 7NG, UK; 3 Medical Research Council Cognition and Brain Sciences Unit, 15 Chaucer Road, Cambridge CB2 7EF, UK; 4 Division of Psychology, Department of Clinical Neuroscience, Karolinska Institutet, Stockholm, Sweden; 5 Department of Psychology and Counselling, Newman University, Birmingham B32 3NT, UK

The current letter by Mole (2016) highlights three recent papers from our laboratory that have attempted to investigate how the initial processing of intrusively remembered episodes leads to their formation (Bourne *et al.* 2013; Clark *et al.* 2014, 2016). These intrusively remembered episodes are from films with traumatic content. Intrusive memories (referred to as flashbacks in Bourne *et al.* 2013) are ones which come to mind *unbidden*, and have particular relevance to post-traumatic stress disorder (PTSD). Mole (2016) suggests that one of the subanalyses performed in Bourne *et al.* (2013) is methodologically flawed and questions some of our subsequent interpretations. While there are elements of the critique we accept, we would like to refute some of the inferences drawn by Mole (2016). In particular, we argue that the main conclusions of our papers are not affected by the ‘methodological flaw’ raised.

The main aim of Bourne *et al.* (2013) and the subsequent papers was to investigate the neural basis of intrusive memory encoding of experimental trauma, hypothesizing that the later occurrence of an intrusive memory would be determined by the neural activity during the original encoding. Results from our three papers support this notion: first, by identifying and subsequently replicating a widespread ‘neural signature’ at the time of viewing (encoding) scenes that later became intrusive (Bourne *et al.* 2013; Clark *et al.* 2016); and second, by using machine learning to predict the occurrence of later intrusive memories solely from the brain imaging data at encoding (Clark *et al.* 2014).

The ‘methodological flaw’ eloquently raised by Mole (2016) relates to a secondary analysis that was performed to investigate potential alternative explanations for our findings; namely, that emotional processing or physiological arousal could completely explain the pattern of brain activation observed (Bourne *et al.* 2013, online Supplementary materials). Mole (2016) specifically critiques our attempt to control for the subjective emotional experience of different scene types. Participants provided retrospective (1-week post-film) ratings of the emotionality of each scene on a 10-point scale. These ratings were then used as covariates in a secondary analysis of the functional magnetic resonance imaging (fMRI) data. The limitation that Mole (2016) raises concerns the mixture of coarse-grained (subjective emotional rating) and fine-grained [fMRI blood oxygen level-dependent (BOLD) signal] measurements. We accept this potential limitation, but note that the BOLD response itself is a relatively course measure of relative changes in brain activity (e.g. Raichle, 1998). Further, we support Mole's (2016) point that a persistence of activations associated with intrusive memories even following covariation of the coarse-grained emotional response does not mean that these activations could not be mediated by finer-grained measurements of emotion (or emotion in general). Indeed, Bourne *et al.* (2013) state that ‘intense emotional reaction may be a necessary condition for flashback formation’ (p. 1529). However, we question the extent to which this could be described as a ‘flaw’ in our data, interpretations and conclusions. This limitation has no bearing on our main analyses nor the interpretations and conclusions that are drawn – namely that there is a specific peri-traumatic pattern of brain activation that predicts intrusive memory formation.

Thus, while Mole (2016) raises interesting potential methodological limitations of our secondary analysis, we disagree with the interpretations and conclusions that Mole draws. Bourne *et al.* (2013) did not claim that the secondary analysis was a mathematical proof, nor that it meant that emotion played no role in intrusive memory encoding. Rather, Bourne *et al.* (2013) used the secondary analysis (heavily caveated by several methodological limitations) together with the region of interest (ROI) analysis to suggest that emotional response did not seem to fully explain the differential processing detected in intrusive-memory encoding. Specifically, the ROI analysis (Bourne *et al.* 2013, p. 1527) provides two key pieces of information: first, many brain regions not associated with emotional processing are implicated (with the acknowledged caveat of the limitations of reverse inference); and second, although the pattern of BOLD response for many brain regions could be compatible with an emotion-mediated process [i.e. Actual (Intrusive) > Potential > Control], crucially the left inferior frontal gyrus and middle temporal gyrus show activation that is not compatible with simple increasing levels of emotionality – activity in these areas is lower during Potential scenes than during both Control and Actual (Intrusive) scenes.

Mole (2016) argues that there are distinct emotion-based and cognitive-based hypotheses for the formation of intrusive memories. He states: ‘Bourne *et al.* suggest that the evidence given in their 2013 study favours hypotheses of this second type’ – referring to a favouring of cognitive processing over emotional processing for the formation of intrusive memories. We strongly disagree. We wrote that ‘although intense emotional reaction may be a necessary condition for flashback formation it appears not to be sufficient’ in Bourne *et al.* (2013; p 1529), by which we intended to suggest that intrusive memoires are not formed solely due to extreme emotion, but also due to a number of other factors. In other words, heighted emotion is necessary, but alone it is not sufficient, requiring the involvement of other cognitive processes for intrusive memory formation.

We continue to argue that both cognitive processing and emotional processing are important for intrusive memory formation – that these are not alternative hypotheses. Peri-traumatic emotional processing has long been highlighted as important for the development of PTSD (e.g. American Psychiatric Association, 2000; Brewin & Holmes, 2003; Brewin, 2014) and for experimentally induced intrusive memories (e.g. Clark & Mackay, 2015; Clark *et al.* 2015). For intrusive/involuntary memories, emotional processing includes negative and positive emotions (Berntsen & Rubin, 2008; Clark *et al.* 2013). Other cognitive process, e.g. pre-trauma scores of depression, anxiety, schizopty and attention, have also been highlighted as important for intrusive memory formation (Steel *et al.* 2008; Verwoerd *et al.* 2008; Clark *et al.* 2015). Unlike Mole (2016), we do not argue that these are distinct hypotheses and explanations. Instead, we suggest the evidence taken together strongly implies that emotional processing is important for intrusive memory formation but that other factors in addition to emotion are also important (as we have argued elsewhere, e.g. Holmes & Bourne, 2008).

While we emphasize the importance of understanding how intrusive memories are first encoded, we also acknowledge the significance of processing at later time points. Understanding processing at the time of experimental trauma is essential for understanding the formation of intrusive memories; for example, performing visuospatial tasks while viewing traumatic footage reduces the frequency of later intrusive memories (Bourne *et al.* 2010); the neural underpinnings during encoding are different for later *intrusively* remembered footage than material that does not intrude (Bourne *et al.* 2013; Clark *et al.* 2014, 2016). However, we welcome investigations of other aspects of intrusive memory recollection and interpretation post-trauma. Indeed, work from our group highlights the importance of the memory (re)consolidation phases after viewing experimental trauma – manipulations post-event using visuospatial tasks can reduce intrusive memory formation (Holmes *et al.* 2010; James *et al.* 2015). Our neuroimaging papers (Bourne *et al.* 2013; Clark *et al.* 2014, 2016) attempt to highlight the importance of the relatively under researched area of peri-traumatic factors in addition to the post-traumatic factors which are more commonly studied.

Mole (2016) finishes his critique highlighting the ‘inherent limitations of affective introspection (Haybron, 2007)’ in relation to the limitations of our coarse-grained analysis. Reliance on self-report measures and introspection without objective measures has long been an issue in understanding human cognition. Physiological measures (e.g. fMRI and heart rate) attempt to overcome some of the limitations of self-report. Interestingly, heart rate was also used as a covariate in Bourne *et al.* (2013) in a separate secondary analysis, with convergent results to both that of the main and emotion covariate analyses. Self-report measures remain a mainstay of psychiatry and psychology, and are predictive of later symptoms of PTSD (e.g. self-report peri-traumatic emotional response and peri-traumatic dissociation; Ozer *et al.* 2003). Until such day a magic device is developed that can provide an objective and fine-grained measure of subjective emotions (and other cognitions), they remain, despite inherent limitations, a valuable part of experimental medicine, experimental psychopathology and clinical studies.

We thank Mole (2016) for raising an interesting discussion in regards to methodological limitations within one of the secondary analyses presented in Bourne *et al.* (2013). In sum, the main aim of the original paper (and our subsequent replication) was to investigate whether the occurrence of an intrusive memory is determined by the neural activity during the original encoding of experimental trauma. While we acknowledge some limitations of our subanalyses testing alternative explanations for our findings, these limitations do not detract from the main findings and conclusions of our papers. Understanding the contributions of heightened memory and emotional processing at the time of traumatic stimuli is critical (but not exclusively so!) for the development, and possible treatment, of intrusive memories in PTSD and elsewhere.
